# Social inequalities in social-emotional problems among preschool children: a population-based study in Sweden

**DOI:** 10.1080/16549716.2022.2147294

**Published:** 2023-02-01

**Authors:** Masoud Vaezghasemi, Anni-Maria Pulkki-Brännström, Marie Lindkvist, Sven-Arne Silfverdal, Wolfgang Lohr, Anneli Ivarsson

**Affiliations:** a Department of Epidemiology and Global Health, Umeå University, Umeå, Sweden; b Department of Clinical Science, Pediatrics, Umeå University, Umeå, Sweden

**Keywords:** Ages and Stages Questionnaires: Social-Emotional (ASQ:SE), mental health, preschool children, social inequality, Population-based

## Abstract

**Background:**

Social-emotional ability is important for overall health and wellbeing in early childhood. Recognizing preschool children in need of extra support, especially those living in unfavourable conditions, can have immediate positive effects on their health and benefit their wellbeing in the long-term.

**Objectives:**

The aim of this study is to investigate whether there are social inequalities in preschool children’s social-emotional problems, and whether inequalities differ between boys and girls.

**Method:**

This study utilized repeated measures from cross-sectional population-based surveys of three-year old children (2014–2018). The final study population comprised of 9,099 children which was 61% of all the eligible children in Västerbotten County during the study period. The Ages and Stages Questionnaires: Social-Emotional (ASQ:SE) 36-month interval was used to measure children’s social-emotional ability. Social inequalities were studied with respect to parents’ income, education, and place of birth, for which data was obtained from Statistics Sweden. Multiple logistic and ordered regressions were used.

**Results:**

Among 3-year-olds, social-emotional problems were more common in the most vulnerable social groups, i.e. parents in the lowest income quintile (OR: 1.45, p < 0.001), parents with education not more than high school (OR: 1.51, p < 0.001), and both parents born outside Sweden (OR: 2.54, p < 0.001). Notably, there was a larger difference in social-emotional problems between the lowest and highest social categories for girls compared to boys. Higher odds of social-emotional problems were associated with boys not living with both parents and girls living in the areas of Skellefteå and Umeå, i.e. more populated geographical areas.

**Conclusion:**

Already at 3-years of age social-emotional problems were more common in children with parents in the most vulnerable social groups. This does not fulfil the ambition of an equitable start in life for every child and might contribute to reproduction of social inequalities across generations.

## Introduction

Children’s social-emotional ability is important for their overall health and wellbeing in childhood and throughout the life course. Social-emotional ability refers to a child’s ability to develop and engage in positive interactions with peers, siblings, parents and other adults, and to effectively regulate emotions within the context of family, community and culture [1–4]. Inadequacies in children’s social and emotional development are increasingly linked to many public health issues such as mental health problems and substance abuse in later life [5]. Healthy development in the early years, especially from birth up to three years of age [6] or until they transition to school [7], provides the building blocks for children to flourish later in life including successful parenting of the next generation.

Children and adolescents (4–18 years of age) who experience socioeconomic disadvantage are two to three times more likely to develop mental health problems [8] and have a poorer cognitive and socioemotional development in Europe [9] and globally [10]. Even among Swedish preschool children (three to five year-olds), factors such as relatively lower parental education and country of origin outside Sweden, have been associated with more behavioural problems as measured by the Strengths and Difficulties Questionnaire (SDQ) [11]. Our previous research demonstrated associations between preschool children’s social-emotional problems and demographic characteristics such as sex, number of siblings, urban/rural residency, and custody arrangements [12], as well as lifestyle habits [13].

Sweden has historically experienced low health disparity [14] and overall population health is improving [15]. However, like many other countries, Sweden is experiencing systematic disparities in health between different social groups with respect to neighbourhood, sex, age and educational background, and in some contexts, socioeconomic disparities are even rising such as for life expectancy [15]. Similarly, whilst most children and adolescents in Sweden report a good standard of health, those living in socially disadvantaged environments are more likely to endure adverse health outcomes during childhood and later in life [16,17]. Despite this, previous research in Sweden and Europe has mainly focused on adults [9,18] with little attention paid to preschool children. However, this is a period during which inequalities in health could be mitigated both for children today and adults in the future [19,20].

Mental health in children and adolescents is a topic of increasing importance and a major public health concern in Sweden [21]. Many studies report a worrying upward trend in the prevalence of mental health problems in school-aged children [22]. Thus, studies which can identify risk factors for mental health problems in adults in Sweden [23–25] are needed, as well as studies which investigate inhibitors to children’s psychological development [26]. In addition, the Swedish public health policy framework calls for ‘good and equal health’ from early life [27,28]. Recognizing children in need of extra support, especially those living in unfavourable conditions, can have immediate positive effects on their health and benefit their wellbeing in the long-term.

The aim of this study is to investigate whether there are social inequalities in social-emotional problems at three years of age, and whether inequalities differ between boys and girls in Sweden. Social inequalities are studied based on parents’ income, education, and place of birth.

## Methods

### Study design and context

This study is part of the Salut Child Health Intervention Programme in Västerbotten County, northern Sweden. The Salut Child Health Intervention Programme was introduced in 2005 and includes health promoting efforts for all parents and children in the county during their regular visits to Child Health Care [29,30]. The most vulnerable and critical period for every child’s development is the first 1000 days of life [31]. Accordingly, a child health visit at 2½-3 years of age has been implemented in the Swedish child health program [32]. At the children’s 3-year health check-ups, parents are encouraged to fill out the Salut questionnaire, in which the Ages and Stages Questionnaire: Social-Emotional (ASQ:SE) [4] is included. The ASQ:SE instrument that is considered to have adequate psychometric properties that includes validity and reliability internationally [33] was introduced to increase parents’ awareness of children’s social and emotional behaviours and to identify both children at risk and parents who may benefit from extra support. We have also studied the internal consistency and unidimensionality of the ASQ-SE among three-year-olds in the Swedish Context using Confirmatory Factor Analysis [34]. In this repeated cross-sectional population-based study, ASQ:SE records from three-year old children in 2014–2018 were linked to national population registers to obtain family socioeconomic and sociodemographic information.

### Study participants

**Figure 1.**
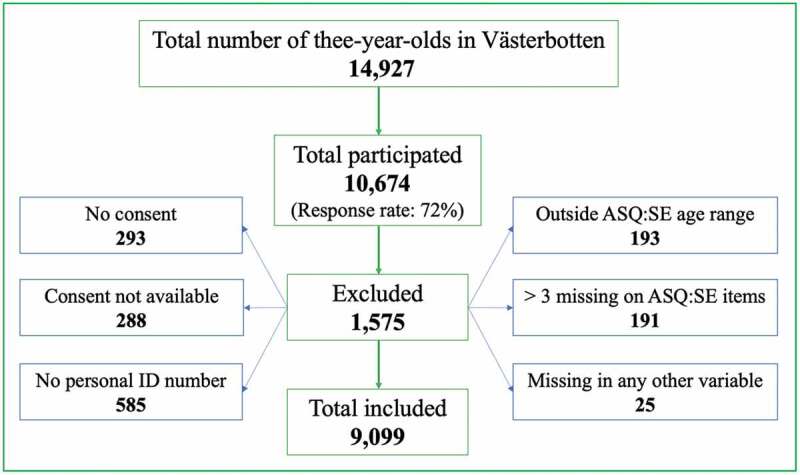
Flowchart showing study participants and exclusion criteria.

Out of total number of 14,927 three-year-olds living in Västerbotten County during the study period [35], 10674 were included in the survey (response rate 72%). Inclusion required that children had to have had a routine three-year check-up in Child Health Care during 2014–2018 and parents had to have responded to the Salut three-year-olds questionnaire. In total 1,575 children (15%) were excluded (Figure 1) for one or more of the following reasons: i) missing informed consent (n = 581); ii) missing or incorrect personal identity number for children (n = 585) which precluded record linkage; iii) the Salut questionnaire, including the ASQ:SE 36-month interval, had been completed when the child’s age was outside the recommended range of 33 to 41 months (n = 193); iv) the ASQ:SE had more than three unanswered questions (n = 191), and v) missing data for any other variable (n = 25). The final study population comprised 9,099 children which was 61% of the population of three-year old children in Västerbotten during the study period.

### The instrument

The Salut questionnaire for three-year-olds included the ASQ:SE 36-month interval in its first edition [4]. The ASQ:SE 36-month interval measures social-emotional developments or abilities specific for this age and also intends to identify children being at risk of social-emotional problems. The original English version of the questionnaire was available; however, most parents used the Swedish version which followed recommended translation principles [36]. Parents responded on a three-point Likert scale (0, 5, or 10 points) indicating how often they perceived that their child had the specific behaviour, and whether that behaviour was a concern for them (5 points).

This analysis used data captured from thirty-one questions excluding the last three open-ended questions. In accordance with the ASQ:SE User’s Guide [33] we replaced up to three missing responses by the average value from all the other responses. Possible scores range from zero to 465 points, whereby zero means no behavioural problem identified on any item, and 465 means a problem was indicated on all items of concern.

### Variables and definitions

#### Outcome variable

The outcome ‘*social-emotional problems’* was defined as being at risk of having ‘problems’ (i.e. above the cut-off) in this study. Although the ASQ:SE focuses on children’s social-emotional developments or abilities, our focus was to identify and address potential social-emotional concerns or problems. Thus, we constructed two binary variables by using the United States cut-off score of 59 recommended by the ASQ:SE User Guide [33], and by using the cut-off score of 50 based on our previous analysis [37]. The latter suggested that a cut-off of 50 may be more relevant in the Swedish context. An ordinal outcome was also constructed comprising three levels i.e. below the cut-off 50, 50 to below 59, or 59 and above.

#### Explanatory variables

Information on parents’ income, education and place of birth was obtained from the National Registers managed by Statistics Sweden and linked to the children’s ASQ:SE information by using the children’s unique personal ID number. The linkage was undertaken by Statistics Sweden, pseudonymised and returned to the study investigators. Parental characteristics used as explanatory variables are defined below:

*Parents’ income* was based on average parental disposable income in the previous three calendar years of every child’s life, i.e. from early pregnancy until every child is two years old. For instance, if a child was three years old in 2014, his/her family income from 2011 to 2013 was used. Disposable income is defined here as the amount of money available to be spent or saved at discretion, after deducting taxes and social security charges. In order to account for inflation, income (in Swedish SEK) was adjusted to a common reporting year (2017) using the Consumer Price Index [38]. The continuous income variable was then categorised into quintiles (highest = first to lowest = fifth).

*Parents’ education* was based on the highest level of education achieved by each parent and categorised into: i) both more than high school; (ii) one more than high school; or (iii) none more than high school.

*Parents’ place of birth* was based on the country where parents were born and categorised into: (i) both born in Sweden; (ii) one born in Sweden; or (iii) both born outside Sweden.

*Custody arrangement* was categorised as: (i) living with both parents; or (ii) not living with both parents.

*Place of residence* was intended to indicate where the family was living at the time of the child’s three-year old check-up. It was divided into well-established parts of the County: (i) Södra Lappland; (ii) Skellefteå; or (iii) Umeå. Södra Lappland is situated in the County’s western area with Lycksele as the most populated municipality (12,228, in 2018), Skellefteå is situated in the northeast with Skellefteå municipality being the most populated (72,467, in 2018), and Umeå is situated in the southeast with Umeå municipality (127,119, in 2018) being the most populated [35].

### Statistical analysis

Predictive margins [39] were used to assess whether the proportion of social-emotional problems differs significantly between and within gender groups across time. This was done by including the interaction between gender and year in a logistic regression model. Since the proportion differed between boys and girls in each set of cross-sectional data, but not among boys and girls across time, we combined the data from 2014–2018 and conducted a pooled analysis for all and also for boys and girls separately. Numbers and percentages were used to present the characteristics of participants. Pearson’s chi-squared test was used to investigate the differences across each characteristic. Two models were applied for multiple logistic regression using the 50 cut-off and the 59 cut-off for defining the outcome variable. We then extended the analysis to consider the proportional odds assumption or the parallel regression assumption by conducting a multiple ordered logistic regression in which the outcome variable was treated as an ordinal variable (below the cut-off 50, 50 to below 59, or 59 and above). Multiple regressions allow to assess the strength of the relationship between several explanatory variables simultaneously and the outcome variable. Odds Ratios (OR) and 95% Confidence Intervals (95% CI) were reported, and a p-value of less than 0.05 was used to denote significant statistical associations. Data were analysed using Stata/SE version 16.0 (StataCorp, College Station, Texas 77,845 US).

## Results

### Characteristics of the study population

A total of 9,099 children, 4,694 (52%) boys and 4,405 (48%) girls were included in the sample. Distribution of social-emotional problems (or being at risk of having ‘problems’) defined by two cut-off scores (≥50 or ≥59), stratified for boys and girls and by social characteristics are presented in Table 1. Among all children, 1,451 (16%) had social-emotional problems when using the cut-off score of ≥50. Using the higher cut-off (≥59) the corresponding number was 861 children (9%). Boys were more likely to have social-emotional problems than girls, irrespective of cut-off score used.

**Table 1.** Characteristics of the study population and distribution of the social-emotional problems, measured by Ages and Stages Questionnaires: Social-Emotional (ASQ:SE), using the cut-off scores of ≥50 and ≥59.

		ASQ:SE Score					
	50 OR ABOVE				59 OR ABOVE		
	All N (%)	All N (%)	Boys N (%)	Girls N (%)	All N (%)	Boys N (%)	Girls N (%)
**Study participants**	9099 (100)	1451 (16)	952 (20)	499 (11)	861 (9)	589 (13)	272 (6)
**Characteristics**							
**Parents’ income**							
Highest quintile 1	1827 (20)	237 (13)	154 (17)	83 (9)	136 (7)	95 (11)	41 (4)
2	1818 (20)	264 (15)	166 (18)	98 (11)	154 (8)	102 (11)	52 (6)
3	1811 (20)	263 (15)	187 (19)	76 (9)	139 (8)	100 (10)	39 (5)
4	1818 (20)	271 (15)	184 (20)	87 (10)	162 (9)	117 (12)	45 (5)
Lowest quintile 5	1819 (20)	414 (23)	259 (27)	155 (18)	268 (15)	173 (18)	95 (11)
**Parents’ education**							
Both more than high school	3162 (35)	426 (13)	281 (17)	145 (9)	244 (8)	173 (11)	71 (5)
One more than high school	2948 (32)	443 (15)	304 (20)	139 (10)	254 (9)	188 (12)	66 (5)
None more than high school	2949 (33)	574 (19)	362 (23)	212 (15)	360 (12)	226 (15)	134 (10)
**Parents’ place of birth**							
Both born in Sweden	7689 (85)	1108 (14)	734 (19)	374 (10)	648 (8)	441 (11)	207 (6)
One born in Sweden	986 (11)	216 (22)	141 (27)	75 (16)	131 (13)	96 (18)	35 (8)
Both born outside Sweden	328 (4)	109 (33)	67 (37)	42 (29)	72 (22)	47 (26)	25 (17)
**Custody arrangement**							
Living with both parents	8222 (92)	1252 (15)	821 (19)	431 (11)	724 (9)	497 (12)	227 (6)
Not living with both parents	706 (8)	159 (23)	105 (28)	54 (17)	107 (15)	73 (19)	34 (10)
**Place of residence**							
Södra Lappland	1116 (12)	160 (14)	115 (20)	45 (8)	100 (9)	76 (13)	24 (4)
Skellefteå	2395 (26)	396 (17)	246 (20)	150 (13)	234 (10)	147 (12)	87 (8)
Umeå	5587 (61)	895 (16)	591 (21)	304 (11)	527 (9)	366 (13)	161 (6)

The differences in social-emotional problems (cut-offs ≥50 and ≥59) between boys and girls were all statistically significant. Place of residence was significant only for girls.

One-third of children had parents with low educational attainment (i.e. no more than high school). Four percent had both parents born outside Sweden, while 11% had one parent born outside Sweden. Eight percent were living without one of their parent(s). A total of 61% resided in the Umeå area, 26% in the Skellefteå area, and 12% in the Södra Lapland area.

Across all social characteristics, the proportion of children having social-emotional problems was higher in the most vulnerable social groups, i.e. parents in the lowest income quintile, parents with education not more than high school, and both parents born outside Sweden. Furthermore, within each category, a higher proportion of boys had social-emotional problems. These patterns were consistent for both ASQ:SE cut-offs (i.e. ≥50 and ≥59). For example, using the cut-off of ≥50, 23% of children with parents in the lowest income quintile compared to 13% in the highest quintile had problems; among children with both parents born outside Sweden, 37% of boys and 29% of girls had problems. There was no observable pattern for place of residence. However, after stratifying by sex, girls in Södra Lappland had lower social-emotional problems compared to those in Skellefteå and Umeå. In addition, within each area more boys than girls had social-emotional problems.

In addition, the differences between the lowest and highest social groups were larger for girls compared to boys. For instance, considering cut-off ≥59, 11% of boys with social-emotional problems were in highest income quintile and 18% in the lowest, while for girls it was 4% and 11%.

### Association between social-emotional problems and social characteristics

Table 2 presents two series of multiple logistic regressions, one for each ASQ:SE cut-off (≥50 and ≥59) for all children and separately for boys and girls. Girls were significantly less likely to have social-emotional problems compared with boys irrespective of the cut-off used. Across all social characteristics, being in the lowest category (in respect of relative disadvantage) was significantly associated with having social-emotional problems.

**Table 2.** Associations between social-emotional problems, measured by Ages and Stages Questionnaires: Social-Emotional (ASQ:SE), and social characteristics using multiple logistic regression with the cut-off scores of ≥ 50 or ≥59, respectively.

	ASQ:SE Score					
	50 OR ABOVE			59 OR ABOVE		
	All N=8823	Boys N=4534	Girls N=4289	All N=8823	Boys N=4534	Girls N=4289
	OR	OR	OR	OR	OR	OR
**Parents’ income**						
Highest quintile 1	1	1	1	1	1	1
2	1.10	1.03	1.22	1.11	1.04	1.29
3	1.04	1.07	0.98	0.92	0.88	0.99
4	1.04	1.06	1.00	1.05	1.07	1.00
Lowest quintile 5	1.45***	1.35*	1.63**	1.48**	1.32	1.83**
**Parents’ education**						
Both more than high school	1	1	1	1	1	1
One more than high school	1.15	1.21*	1.04	1.14	1.22	0.99
None more than high school	1.50***	1.43***	1.62***	1.58***	1.39**	2.03***
**Parents’ place of birth**						
Both born in Sweden	1	1	1	1	1	1
One born in Sweden	1.45***	1.41**	1.55**	1.38**	1.52**	1.16
Both born outside Sweden	2.54***	2.32***	3.01***	2.49***	2.40***	2.83***
**Custody arrangement**						
Living with both parents	1	1	1	1	1	1
Not living with both parents	1.28*	1.30*	1.24	1.44**	1.43*	1.45
**Place of residence**						
Södra Lappland	1	1	1	1	1	1
Skellefteå	1.32**	1.11	1.82**	1.22	0.98	1.89**
Umeå	1.34**	1.21	1.67**	1.27*	1.11	1.72*
**Gender**						
Girls	1			1		
Boys	2.00***	—–	—–	2.21***	—–	—–

* *p*<.05; ** *p*<.01; *** *p*<.001. OR: Odds Ratio.

Observing the regressions for girls and boys separately, the results were more mixed for custody arrangement and place of residence. Under both cut-offs, for boys, not living with both parents was associated with social-emotional problems, whereas for girls, there was a significant association between place of residence and social-emotional problems. For instance, girls living in Skellefteå and Umeå had higher odds of social-emotional problems compared to those living in Södra Lappland. Regarding parents’ income and education, the most disadvantaged category was associated with about 1.5 times higher odds of having problems compared to the least disadvantaged, except that when using cut-off ≥59, the association between parent’s income and social-emotional problem was not statistically significant for boys. Having one parent not born in Sweden was associated with higher odds of the same magnitude (about 1.5), whereas an even larger association (about 2.5) was found if both parents were born outside Sweden. These associations were somewhat larger for girls than for boys, except that when using the cut-off of ≥59, having one parent born outside Sweden was not associated with higher odds of social-emotional problems.

**Figure 2.**
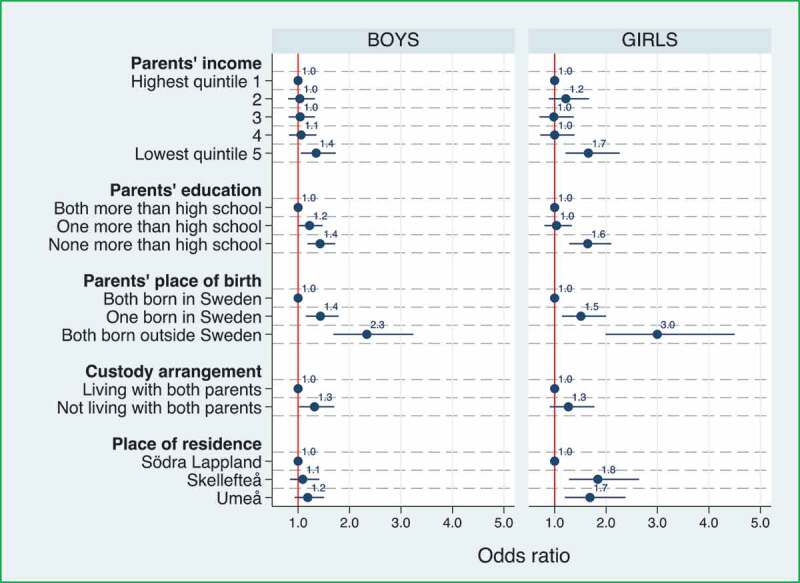
Associations between social-emotional problems, measured by Ages and Stages Questionnaires: Social-Emotional (ASQ:SE), and social characteristics. Results of a multiple ordered logistic regression where the odds ratios describe the relationship between the ASQ:SE levels; below the cut-off 50, 50 to below 59, or 59 and above. The dots present Odds Ratios (OR) and the horizontal lines crossing the dots illustrate the 95% Confidence Intervals (CI).

The same pattern is seen in the ordered logistic regression. Figure 2 and Table 3 present the results of ordered logistic regressions where the ORs describe the relationship between the three ASQ:SE levels constructed; below the cut-off 50, 50 to below 59, or 59 and above. Consistent with the above results, being in the lowest income quintile, having low-educated parents, or one or both parents born outside Sweden, were factors associated with about 1.5 times higher odds of being above either ASQ:SE cut-off. Girls were significantly less likely than boys to be above either cut-off (Table 3). The highest OR is observed for girls with both parents born outside Sweden. This group had three times the odds of being above either cut-off compared to girls with both parents born in Sweden. Not living with both parents was associated with 1.3 times higher odds of being above either cut-off for boys. Finally, for girls, living in the Umeå or Skellefteå areas, compared to Södra Lappland, was associated with 1.7 and 1.8 times higher odds of having social-emotional problems, respectively.

**Table 3.** Associations between social-emotional problems, measured by Ages and Stages Questionnaires: Social-Emotional (ASQ:SE), and social characteristics. Results of a multiple ordered logistic regression.

	ASQ:SE Score		
	All N=8823	Boys N=4534	Girls N=4289
**Characteristics**	OR	OR	OR
**Parents’ income**			
Highest quintile 1	1	1	1
2	1.10	1.04	1.22
3	1.03	1.04	0.98
4	1.04	1.06	1.00
Lowest quintile 5	1.45***	1.35*	1.66**
**Parents’ education**			
Both more than high school	1	1	1
One more than high school	1.15	1.22*	1.04
None more than high school	1.51***	1.43***	1.64***
**Parents’ place of birth**			
Both born in Sweden	1	1	1
One born in Sweden	1.45***	1.43**	1.51**
Both born outside Sweden	2.54***	2.34***	3.00***
**Custody arrangement**			
Living with both parents	1	1	1
Not living with both parents	1.30*	1.32*	1.27
**Place of residence**			
Södra Lappland	1	1	1
Skellefteå	1.31*	1.09	1.84***
Umeå	1.33**	1.19	1.69**
**Gender**			
Girls	1		
Boys	2.00***	—–	—–

* *p*<0.05; ** *p*<0.01; *** *p*<0.001. OR: Odds Ratio.

## Discussion

### Main findings

Our study has revealed social differences in social-emotional problems, as measured by ASQ:SE, in three-year old children in Sweden. Children in vulnerable social categories were more likely to have social-emotional problems. Thus, the lowest categories of parental income and parental education were associated with a similar sized increased likelihood of social-emotional problems, with this likelihood being even higher for children with both parents born outside Sweden. Boys were more likely to have social-emotional problems compared to girls, across all social groups. However, there was a larger difference in social-emotional problems between the lowest and highest social category for girls compared to boys. Higher odds of social-emotional problems were observed among boys not living with both parents and girls living in the areas of Skellefteå and Umeå, i.e. more populated geographical areas.

### General discussion

Our results support previous studies showing that children with vulnerable parental social status are at higher risk of mental health problems. While most of this literature concerns older children [8,40], some results for preschool children support our findings. For example, a large community sample from Sweden found that lower parental education and having parents born abroad were factors associated with more behavioural problems among three- to five-year-olds [11]. Yet few previous studies have investigated the distribution of mental health related problems in this age group at the population level. In Uruguay, data from nationally representative studies found a socioeconomic gradient by household income quintile using the same instrument as our current study [41]. They also showed a socioeconomic gradient for maternal education using another instrument, the Child Behaviour Checklist [42]. Whereas in Uruguay, there appears to be a gradient even across the band of more advantaged groups, our results show significantly higher odds only for parents in the lowest income quintile and lowest educational category.

### Strengths and limitations

A major strength of this study is that the results are population-based with a high participation rate (about 72%). Utilising the ASQ:SE, we provided a comprehensive analysis of social differences in preschool children’s development and social-emotional problems in northern Sweden. Our results reveal that patterns of social inequalities differ between boys and girls in this age group, which has rarely been investigated in previous studies. It was possible to study several socioeconomic and demographic characteristics because of access to highly reliable individual-level national register data. A particular strength is that we do not rely on self-reports for income; instead, register data on parents’ disposable income is used for the period from birth up to child aged three years. Furthermore, our results are computed using both the manual-recommended ASQ:SE cut-off to allow for international comparison but also a lower cut-off, which was previously found to be optimal in a Swedish context [37]. We found similar results for both ASQ:SE cut-offs with respect to social-emotional problems for boys and girls and in relation to vulnerable social groups, i.e. parents in the lowest income quintile, parents only having high school, and both parents born outside Sweden. These findings strengthen the case for a Swedish cut-off to both identify children at risk of social-emotional problems and also to monitor social inequalities.

Our findings regarding parent’s place of birth need to be interpreted in light of the role that language barriers and cultural background may play in data collection and parental responses. The ASQ:SE questions require a relatively high level of language proficiency, and parents with weak Swedish and English were probably less likely to complete the survey. Therefore, it is likely that differences in completion rates result in underestimating the observed inequalities. We did not have information on parents’ age and for migrant parents, length of stay in Sweden. While it is unlikely that lack of this information will alter our results, they are particularly important for observing heterogeneity within social groups. Furthermore, very little is known about the extent to which migrant and non-migrant parents on the one hand, and different groups of migrant parents on the other, may make systematically different assessments of their preschool child’s social-emotional behaviour. We posit that there are potential cultural differences in parental responses as found in girls with both parents born outside Sweden. Further investigations of health inequalities, using both quantitative and qualitative methods, are needed across children with migrant backgrounds. This is an important area for future research. In addition, there may be interaction effects between different socioeconomic variables or between demographic, socioeconomic and children’s lifestyle indicators, which was not considered in the current study. Future research should go beyond unidimensional social gradients toward more complex interactions between axes of social differentiation, e.g. using an intersectional approach [43–45].

### Policy and practice implications

Our findings on social inequalities complement previous results on the prevalence of social-emotional problems among preschool children in Sweden [37]. Together this body of knowledge strongly suggests that policies and interventions for mental health promotion are needed in early life. Even though Sweden’s income per capita is high in an international comparison, about 9% of all children (6% in Västerbotten County) live in absolute poverty [46] meaning that household income is insufficient to cover essential expenses. Our results imply that income support for the poorest families is essential, but actions must also address other types of social disadvantage, particularly parents’ lack of high school education and being born outside Sweden. The future outlook for equitable health appears particularly challenging when inequalities re-emerge across and within subsequent generations. This reproduction of social disparities reduces opportunities for social mobility within different population groups throughout the life course [47]. To break the ‘vicious cycle of disadvantage’ [19], ‘equity from the start’ [20] should be an important element of any endeavour to improve health and reduce health inequalities. This underscores a realignment of equity-in-health research specifically towards early life as a ‘powerful equalizer’ [48].

## Conclusion

Early and persistent exposure to socioeconomic disadvantage may impair children’s own health and wellbeing, and further contribute to reproduction of social disparities across generations. Preschool children’s social-emotional problems being strongly associated with their parents’ social status should be of interest to policy makers aiming to reduce inequalities in child health in Sweden.

## Data Availability

The datasets presented in this article are not readily available because Region Västerbotten originally collected the data for a child health survey (https://www.regionvasterbotten.se/salut). We accessed data for the present study after approval from both the Region Västerbotten and the Ethical Vetting Board. The data are not publicly available but access for replication analyses is possible. Requests to access the datasets should be directed to https://www.regionvasterbotten.se/salut.

## Paper context

While most of the research on health inequalities focuses on adults, we investigated social inequalities in preschool children’s social-emotional problems. Already at 3-years of age social-emotional problems were more common in children with parents in the most vulnerable social groups. This does not fulfil the ambition of an equitable start in life for every child and might contribute to reproduction of social inequalities across generations.
